# Exploring the potential of *Weissella paramesenteroides* UFTM 2.6.1 in disrupting quorum sensing and attenuating virulence in *Listeria monocytogenes*

**DOI:** 10.3389/fmicb.2025.1601203

**Published:** 2025-06-18

**Authors:** Isabela Sguilla Rotta, Sthefânia Dalva da Cunha Rezende, Hugo Felix Perini, Marcos Vinicius da Silva, Felipe Alves de Almeida, Uelinton Manoel Pinto, Alessandra Barbosa Ferreira Machado, Aline Dias Paiva

**Affiliations:** ^1^Department of Microbiology, Immunology and Parasitology, Federal University of Triângulo Mineiro, Uberaba, Brazil; ^2^Department of Education in Science, Mathematics and Technology, Federal University of Triângulo Mineiro, Uberaba, Brazil; ^3^Department of Microbiology, Institute of Biotechnology Applied to Agriculture, Federal University of Viçosa, Viçosa, Brazil; ^4^Department of Food and Experimental Nutrition, Food Research Center, São Paulo University, São Paulo, Brazil; ^5^Department of Parasitology, Microbiology and Immunology, Federal University of Juiz de Fora, Juiz de Fora, Brazil

**Keywords:** biofilm, foodborne pathogen, lactic acid bacteria, probiotic, quorum quenching

## Abstract

**Introduction:**

*Weissella paramesenteroides* UFTM 2.6.1, isolated from unpasteurized milk, is a potentially probiotic strain exhibiting desirable properties previously demonstrated in vitro, along with a confirmed safe and promising genetic profile based on whole-genome analysis. Due to the limited research on the ability of *Weissella* species to synthesize compounds with anti-quorum sensing activity, this study aimed to investigate the potential of *W. paramesenteroides* UFTM 2.6.1 to disrupt quorum sensing (QS) signaling and attenuate the virulence of *Listeria monocytogenes*, an important foodborne pathogen responsible for the zoonotic disease listeriosis.

**Methods:**

The effects of *W. paramesenteroides* cell-free supernatant (*Wp*-CFS) were evaluated on the growth, biofilm formation, motility, and expression of QS- and virulence-related genes in *L. monocytogenes*.

**Results:**

*Wp*-CFS exhibited bacteriostatic activity against L. monocytogenes strains isolated from food and food processing environments (*n* = 21). Additionally, it consistently reduced biofilm formation and swarming motility, two well-known QS-regulated phenotypes in *L. monocytogenes*. Exposure to *Wp*-CFS (0.25x MIC; 7.81 mg/mL), at 28°C for 24 h, significantly downregulated the relative expression of the genes *luxS, agrA, flaA, motA, motB*, and *degU*, whereas the genes *sigB*, and *prfA* were upregulated.

**Conclusion:**

This study represents the first report demonstrating the production of compounds by W. paramesenteroides aimed at disrupting the QS system of *L. monocytogenes*, offering novel insights into alternative approaches to attenuate pathogen virulence without relying on traditional antimicrobials.

## 1 Introduction

The genus *Weissella* belongs to the group of bacteria known as lactic acid bacteria (LAB). The genus was first designated in 1993 after taxonomic studies on atypical *Leuconostoc*-like microorganisms. Bacteria assigned to the genus *Weissella* are commonly found in a variety of environments, including the gastrointestinal tract of humans and animals, plant-associated microbiomes, and fermented foods. They are known for their versatility in surviving different environmental conditions, such as varying pH and temperature levels (Collins et al., 1993). Some *Weissella* strains have been recognized for their probiotic potential, they can also inhibit the growth of bacterial pathogens, modulate gut microbiota, and immune responses (Fhoula et al., 2013). Taken together, these characteristics make *Weissella* an important genus in the field of food microbiology and human health. Among the species of the genus, *Weissella confusa, Weissella cibaria*, and *Weissella paramesenteroides* are the most widely studied (Fusco et al., 2023; Liu et al., 2024; Singh et al., 2024).

*W. paramesenteroides* UFTM 2.6.1, previously isolated from unpasteurized milk by our research group, shows the ability to survive in the presence of bile salts and acidic pH, and exhibits antagonistic properties against spoilage and pathogenic bacteria (Rotta et al., 2020). Recently, the complete genome sequencing of this strain uncovered genes related to probiotic properties, as well as absence of antimicrobial resistance determinants and other virulence genes, revealing a promising safety profile (Rocha et al., 2024).

*Listeria monocytogenes* is a well-known foodborne pathogen, notorious for its ability to withstand stress conditions. It can withstand various treatments commonly used in the food processing environment to control microbial growth, including heat, acidification, salt addition, preservatives, sanitizers, and high hydrostatic pressure. In addition, *L. monocytogenes* is capable of persisting in the environment for extended periods, largely due to its ability to form biofilms (Wiktorczyk-Kapischke et al., 2023; Cheng et al., 2023; Ribeiro et al., 2023; Tuytschaever et al., 2023). Biofilms are defined as aggregates of microbial cells adhered to solid surfaces and embedded in an extracellular polymeric matrix. Within biofilms, microbial cells exhibit increased resistance to sanitizers, detergents, and antimicrobials, and may also express specific virulence genes (Poimenidou et al., 2016; Gemmell et al., 2022).

Biofilm formation is intricately linked to quorum sensing (QS), an inter- and intraspecies communication mechanism that enables bacteria to assess population density through the release and detection of signaling molecules, known as autoinducers (AI). Upon reaching a critical concentration (threshold level), these AIs activate a coordinated response among microbial cells, leading to changes in gene expression. In many pathogens, including *L. monocytogenes*, QS systems are essential for the regulation of key virulence factors, such as biofilm formation, resilience to environmental stress, motility, and resistance to antimicrobial agents (Rieu et al., 2007; Brackman and Coenye, 2015).

Two main QS systems have been described in *L. monocytogenes*: the Agr-like system and the LuxS/AI-2 system. The Agr-like system, which is homologous to the Agr system found in *Staphylococcus aureus*, is used for intraspecies communication, and regulates the expression of genes involved in virulence and stress response (Skandamis and Nychas, 2012; Kocot and Olszewska, 2017). On the other hand, the LuxS/AI-2 system, which is more conserved across different bacterial species, mediates interspecies communication and influences biofilm formation and motility (Belval et al., 2006; Sela et al., 2006; Li et al., 2021; Yu et al., 2022). In this context, disrupting bacterial communication could offer a potential strategy to attenuate *L. monocytogenes* virulence (Marques et al., 2024).

Unlike bactericidal approaches, QS-targeting molecules, also named quorum-quenching (QQ) molecules, do not kill the target bacteria but instead they interfere with the signaling pathways that coordinate bacterial population behaviors. The QS mechanism can be inhibited by: (I) inhibition of AIs synthesis; (II) inhibition of AI secretion and transport; (III) degradation of AIs using either catalytic antibodies, such as abzymes or enzymes, such as lactonases, acylase, hydrolase, and oxidoreductase; (IV) sequestration of AIs using, for example, antibodies against AIs; (V) antibodies that “cover” and therefore block AI receptors; (VI) antagonists of AIs, such as chemical compounds; (VII) inhibition of targets downstream of the binding of the AI to the receptor; and (VIII) post-transcriptional regulation of QS genes via small regulatory RNAs (sRNAs) (Almeida et al., 2023). Marques et al. (2024) showed that proteins potentially secreted by *Lacticaseibacillus rhamnosus* GG and *Lactobacillus acidophilus* NCFM, two probiotic LAB, have the ability to *in silico* bind to the QS proteins Agr, AgrB and AgrC of *L. monocytogenes*.

Considering the variety of bioactive compounds synthesized by members of the *Weissella* genus, we hypothesized that *W. paramesenteroides* UFTM 2.6.1 could be a source of QQ molecules to disrupt the QS mechanisms in *L. monocytogenes*, attenuating the virulence of this important pathogen. By elucidating this interaction, we aim to provide insights into a novel strategy to reduce the virulence of *L. monocytogenes* without relying on bactericidal approaches.

## 2 Materials and methods

### 2.1 Microorganisms and growth conditions

*W. paramesenteroides* UFTM 2.6.1 was grown in Man-Rogosa-Sharpe (MRS) broth (Kasvi, Spain), at 37°C for 18–24 h, under microaerophilic conditions (Rotta et al., 2020). *L. monocytogenes* ATCC 19112 and 20 strains of *L. monocytogenes* isolated from different food and food processing sectors were donated by the *Listeria* Collection (CLIST) of the Bacterial Zoonoses Laboratory at the Oswaldo Cruz Institute (LABZOO/Fiocruz) (Table 1). *L. monocytogenes* strains were grown in Brain Heart Infusion (BHI) broth (Himedia, India), at 28 or 37°C (depending on the experiment), for 18–24 h, in aerobiosis. Bacteria were kept preserved at −20°C in 20% glycerol-containing BHI broth.

**Table 1:** *Listeria monocytogenes* strains: code, serotypes, year of isolation, sources and specimens.

*L. monocytogenes*	Serotype	Year of isolation	Source	Specimen
706	*4b*	2011	Food	Yakisoba
1018	*4b*	2011	Food	Mini pizza
1071	*4b*	2009	Food	Powdered milk
3492	*4b*	2013	Environment	Meat tenderizer
3803	*4bb*	2014	Food	Organic vegetables (Beetroot)
3833	*1/2b*	2014	Environment	Industrial floor
3837	*4b*	2014	Environment	Drain processing room
3839	*1/2b*	2014	Environment	Drain processing room
3992	*1/2b*	2015	Food	Chicken liver pate
4001	*1/2c*	2015	Food	Beef
4107	*1/2a*	2015	Food	Temaki Philadelphia
4251	*1/2b*	2016	Food	Packaged chicken thigh
4313	*1/2b*	2016	Environment	Dough preparation drain swab
4330	*1/2b*	2016	Environment	Slicer argentine beld swab
4449	*1/2a*	2017	Environment	Boards and knives swab
4455	*1/2a*	2017	Food	Frozen chicken carcass
4484	*1/2c*	2017	Food	Frozen chicken meat cuts
4506	*4b*	2017	Environment	Table with mats
4511	*1/2b*	2017	Environment	Roll
4631	*1/2b*	2018	Food	Ready-to-eat processed meat products

### 2.2 Antagonistic activity against *Listeria monocytogenes* strains

The screening of antagonistic activity against *L. monocytogenes* strains was performed using the agar overlay method (Booth et al., 1977). Briefly, colonies of *W. paramesenteroides* UFTM 2.6.1 grown on MRS agar were overlaid with semi-solid BHI agar (0.75% agar) containing 10^6^ CFU/mL of each *L. monocytogenes* strain separately. The plates were incubated overnight under aerobic conditions at 37°C. Anti-*Listeria* activity was determined by the presence of zones of inhibition (>6 mm in diameter) of *L. monocytogenes* growth around the colonies of *W. paramesenteroides* UFTM 2.6.1.

### 2.3 Cell-free supernatant obtention and evaluation of anti-*Listeria* activity

Cell-free supernatant (CFS) was obtained by centrifuging stationary-phase *W. paramesenteroides* UFTM 2.6.1 cultures (12,500 rpm, 15 min) and filtering the supernatant through a 0.22 μm filter. CFS was lyophilized, suspended in saline solution (0.85%) to a final concentration of 500 mg/mL, referred to as *Wp*-CFS. The *Wp*-CFS was stored at −20°C until use.

The inhibitory activity of *Wp*-CFS was evaluated using the micro-broth dilution technique according to the Clinical and Laboratory Standards Institute (CLSI) guidelines (CLSI, 2023). Aliquots of 50 μL of *Wp*-CFS and its serial dilutions (twofold increments) were added to 96-well plates containing 50 μL of BHI broth (2x concentrated). Suspensions of stationary-phase *L. monocytogenes* cultures containing 10^6^ CFU/mL (10 μL) were added to the microplates, and incubated at 37°C for 18 h under aerobic conditions. Uninoculated BHI broth was used as negative control, and uninoculated BHI broth was used as negative control, and BHI broth inoculated only with *L. monocytogenes* was used as positive control. Saline solution was included as an additional control. Minimum inhibitory concentration (MIC) was defined as the lowest concentration at which bacterial growth was completely inhibited visually. Sub-MICs were defined as concentration of the antimicrobial agent below the MIC value, where the agent did not inhibit bacterial growth but could still influence other bacterial processes (CLSI, 2023).

The minimum bactericidal concentration (MBC) for the *Wp*-CFS was determined after taking aliquots (5 μL) from the wells in the microplates showing no growth after MIC determination and inoculated onto the surface of BHI agar plates (CLSI, 2023). After incubation for 48 h at 37°C, the lowest concentration of *Wp*-CFS at which no bacterial colonies were formed was considered as MBC (Rodríguez-Melcón et al., 2022).

### 2.4 Time-kill kinetics study

Time-kill kinetic studies were performed in 1 mL of BHI broth inoculated with logarithmically grown *L. monocytogenes* to yield a final concentration of 5.0 × 10^6^ CFU/mL, in the presence of different concentrations of *Wp*-CFS ranging from 0.25 to 4x MIC. All the flasks were incubated at 37°C and samples of 0.1 mL were taken at 0, 3, 6, 9, 12, 24, 48, and 72 h post-inoculation. Serial tenfold dilutions were prepared in sterile saline and used to determine CFU/mL. Time-kill curve was constructed by plotting log10 of CFU/mL versus time. Bactericidal activity was defined as a decrease of 99.99% (≥3 log10) in CFU/mL compared to the initial inoculum. Bacteriostatic activity was defined as maintenance of the original inoculum level or decrease of less than 99.99% (<3 log10) in CFU/mL compared to the initial sample (Petersen et al., 2007).

### 2.5 *Listeria monocytogenes* biofilm formation

All 21 *L. monocytogenes* strains were tested to determine their biofilm-forming ability on polystyrene microplates at 28°C (Guilbaud et al., 2015). Overnight cultures grown in BHI medium were adjusted to 0.5 McFarland turbidity standard, and 10 μL aliquots were transferred to a 96-well polystyrene microplate containing 100 μL of BHI broth. After incubation for 72 h at 28°C, the microplates were washed three times with sterile distilled water to remove planktonic cells. Crystal violet (0.25%, 150 μL) was added to each well of the microplate, and the plate was kept at room temperature for 30 min. The crystal violet solution was removed, washed with running water and allowed to dry; then ethanol (95%, 150 μL) was added to solubilize the stain. Absorbance was determined at 540 nm using a microplate reader. Experiments were performed in triplicate and repeated in three independent experiments. Mean optical density (OD) of the three wells for each sample was compared with the mean absorbance of negative controls, and the strains were classified in weak, moderate, strong or non-biofilm producers (Stepanóvic et al., 2000). Isolates classified as strong biofilm producers were selected for further experiments.

### 2.6 Anti-biofilm and anti-motility activities of *Weissella paramesenteroides* cell-free supernatant

The effect of *Wp*-CFS in *L. monocytogenes* biofilm formation was evaluated using the crystal violet method (Fan et al., 2018; Liu et al., 2021). The microplates were prepared as described in the biofilm formation assay and different concentrations of *Wp*-CFS (0.25x, 0.5x, and MIC) were added before the incubation of the microplates. After 72 h at 28°C, biofilm formation inhibition rates were calculated using the following formula:

$\text{Inhibition rate}\;(\%)=[\begin{array}{l} 1–\text{OD}540\text{nm}\;(\text{sample})/\\ \text{OD}540\text{nm}\;(\text{positive control}) \end{array}]\times 100$.

*L. monocytogenes* biofilms treated with *Wp*-CFS were also evaluated using fluorescence microscopy and DAPI/PI dual staining. Briefly, the biofilm was prepared as described above was stained with 4′,6-diamidino-2-phenylindole (DAPI) (Sigma, UK; 10 μg/mL, 30 min) and propidium iodide (PI) (Sigma, UK; 10 μg/mL, 10 min). Non-treated biofilms were used as positive control. Images were taken using an inverted fluorescence microscope (Invitrogen EVOS FL) equipped with a 40x phase contrast objective and a Sony™ ICX285AL CCD camera.

Swimming and swarming motilities of *L. monocytogenes* strains were evaluated in semi-solid agar (Pieta et al., 2017; Jiang et al., 2021). Approximately 3 μL of stationary-phase *L. monocytogenes* cultures were inoculated into the center of plates containing culture medium for swimming (10 g/L tryptone, 5 g/L NaCl, and 0.3% agar) or swarming (25 g/L Luria-Bertani, 0.5 g/L glucose, and 0.5% agar), supplemented with sub-inhibitory concentrations of the *Wp*-CFS (0.125x, 0.25x, and 0.5x MIC). After incubation for 48 h at 28°C, the diameter of the motility zones was measured in millimeters (mm). Motility pattern in the absence of CFS was used as a control, and defined as 100% motility.

### 2.7 Influence of *Weissella paramesenteroides*-cell-free supernatant on *Listeria monocytogenes* target gene expression

The effect of *Wp-*CFS on the expression of genes involved in QS mechanism, motility and biofilm formation in *L. monocytogenes* was evaluated by quantitative reverse transcription polymerase chain reaction (RT-qPCR). *L. monocytogenes* was grown in BHI broth in the presence or absence of *Wp-*CFS (0.25x MIC) at 28°C for 24 h. The cultures were centrifuged (13,500 rpm, 5 min) and washed with sterile phosphate-buffered saline (PBS). Total RNA was extracted using Trizol (Sigma-Aldrich, United States) and converted to cDNA using the High-Capacity cDNA Reverse Transcription Kit (Thermo Fisher, United States). Validated and previously published primers were used (Table 2). The *16S rRNA* gene was used as internal control.

**Table 2:** Pairs of primers used in the present study for quantitative reverse transcription polymerase chain reaction (RT-qPCR).

Gene	Primer	Sequences (5′-3′)	References
*16S rRNA*	F R	CCGTCAAGGGACAAGCA GGGAGGCAGCAGTAGGGA	Liu et al. (2021)
*agrA*	F R	GCAAGCAGAAGAACGGATTT CTGTGGCACCGATAAAATGA	Pieta et al. (2017)
*luxS*	F R	AAGCACCTTTTGTGAGACTGG CCGTTAGTGTTGTAGCGATGA	Pieta et al. (2017)
*motA*	F R	TTTTACGGGATGTTTTGGAA TCGCTAAGTTTGTCTGGGTT	Pieta et al. (2017)
*motB*	F R	TTTGCTGACACTTTTACTTGC TCTTGTTCGTTTGCTTCTTTC	Pieta et al. (2017)
*prfA*	F R	AGAAACATCGGTTGGCTATT TTGACCGCAAATAGAGCC	Liu et al. (2021)
*flaA*	F R	GGCTGCTGAAATGTCCGAAA TGCGGTGTTTGGTTTGCTTG	Jiang et al. (2021)
*sigB*	F R	TGGATTGCCGCTTACCAAGAA TCGGGCGATGGACTCTACTA	Jiang et al. (2021)
*degU*	F R	ACGCATAGAGAGTGCGAGGTATT CCCAATTCCGCGGTTACTT	Jiang et al. (2021)

For the RT-qPCR experiments, a reaction mix was prepared using PowerTrack SYBR Green Master Mix (Thermo Fisher, United States). Each reaction contained the following components: 5 μL of PowerTrack SYBR Green Master Mix, 0.5 μL of forward primer, 0.5 μL of reverse primer, 0.25 μL of yellow dye, 1 μL of cDNA (20 ng), and 3.25 μL of nuclease-free water, resulting in a final reaction volume of 10 μL. Amplification was carried out using the StepOnePlus™ Real-Time PCR System (Thermo Fisher, United States). The reaction conditions were as follows: an initial holding stage at 95°C for 2 min, followed by 40 cycles of denaturation at 95°C for 15 s and 60°C for 60 s. At the end of the amplification protocol, a melting curve analysis was also performed to confirm the specificity of each qPCR reaction (excluding any nonspecific amplification), which included 95°C for 15 s, 60°C for 1 min, and 95°C for 15 s. The 2^−ΔΔCt^ method was used to analyze the relative expression of target genes using Ct values, with melting curve analysis confirming product specificity (Shi et al., 2023; Livak and Schmittgen, 2001). All experiments were performed in biological duplicates and experimental triplicates.

### 2.8 Statistical analysis

The statistical analyses were performed using GraphPad Prism 8.0 software (GraphPad Software Inc., La Jolla, CA, United States). Comparisons between treated groups and control groups were analyzed by ANOVA followed by Dunnett’s test for multiple comparisons to determine the influence of the *Wp*-CFS on biofilm formation and motility. Differences were considered statistically significant when *p* < 0.05. For the relative gene expression, the data were analyzed by ANOVA, and when statistically significant differences were identified (*p* < 0.05) in relative expression levels calculated by the 2^−ΔΔCt^ method, the genes were considered upregulated (2^−ΔΔCt^ > 1) or downregulated (2^−ΔΔCt^ < 1) in the presence of *Wp-*CFS.

## 3 Results

### 3.1 Antagonistic activity of *Weissella paramesenteroides* UFTM 2.6.1 against *Listeria monocytogenes* strains

**Figure 1:**
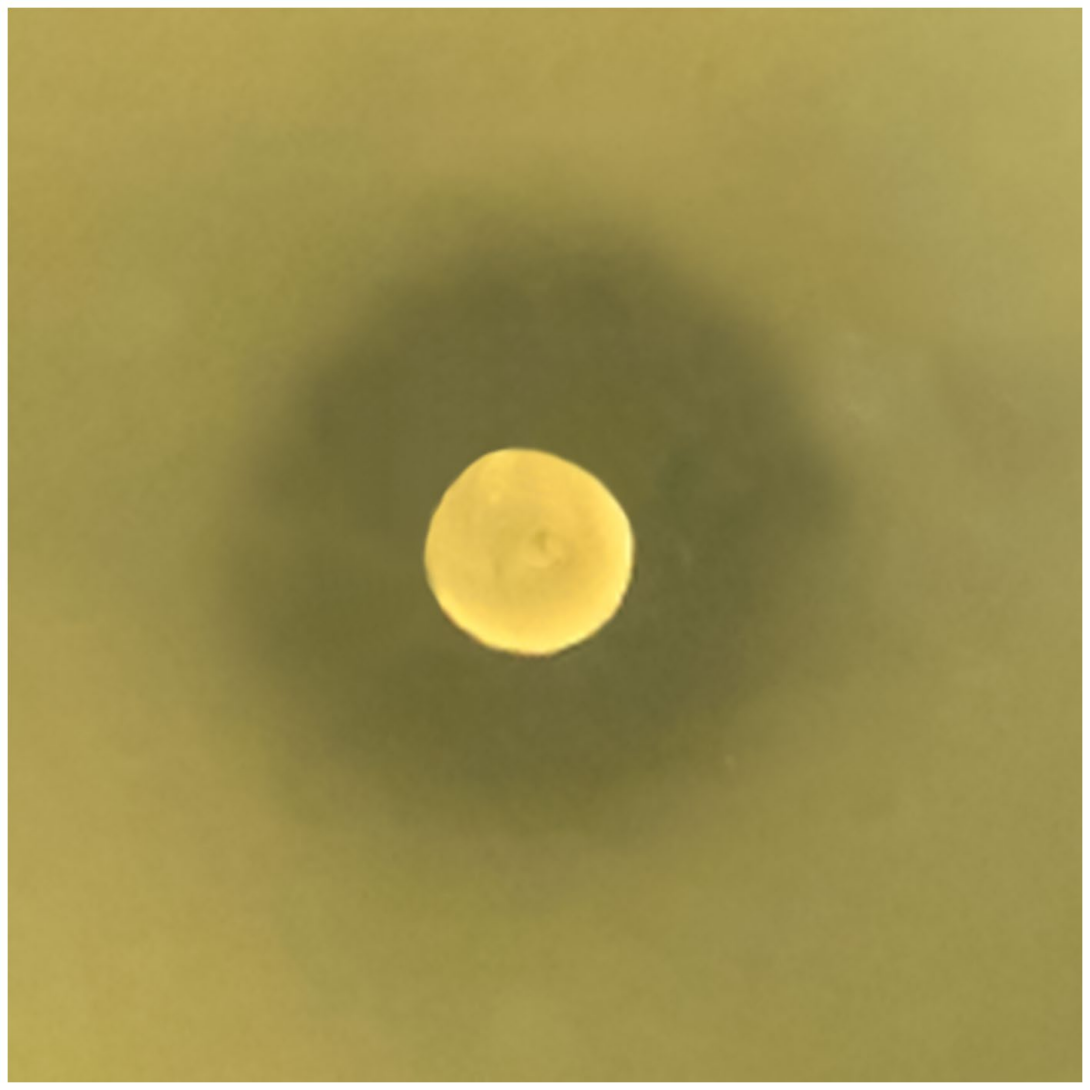
*Weissella paramesenteroides* UFTM 2.6.1 antagonistic activity against *Listeria monocytogenes* on solid medium. The representative image shows the growth inhibition of *L. monocytogenes* strain 4455, with an inhibition zone of 20 mm around the colony of *W. paramesenteroides* UFTM 2.6.1.

*W. paramesenteroides* UFTM 2.6.1 was able to inhibit the growth of *L. monocytogenes* strains on BHI agar overlaid, with inhibition zones ranging from 18 to 22 mm in diameter, suggesting the secretion of anti-*Listeria* compound(s) by this bacterium (Figure 1). The *Wp*-CFS inhibited the growth of all *L. monocytogenes* strains in liquid medium, and a concentration of 31.25 mg/mL was defined as the MIC. The MBC could not be determined, as *L. monocytogenes* formed colonies after exposure to *Wp*-CFS, even at concentrations up to 4x MIC (125.0 mg/mL).

**Figure 2:**
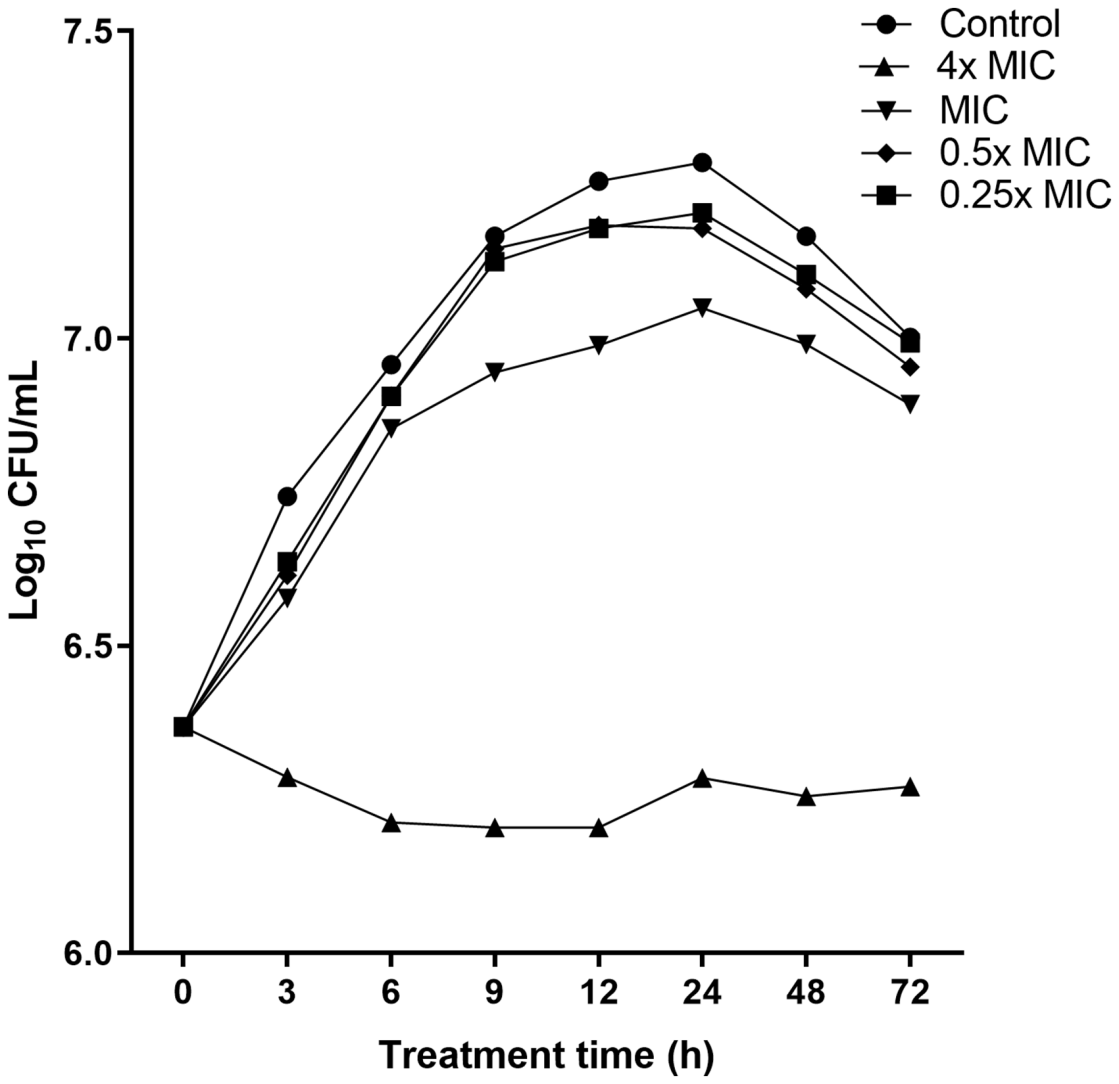
Time-kill curves showing the activity of *Weissella paramesenteroides*-cell-free supernatant (*Wp*-CFS; 4x MIC, MIC, 0.5x MIC, and 0.25x MIC) against *Listeria monocytogenes* strain 4455. *Wp*-CFS was added at time point 0 and monitored until 72 h. Control represents the curve obtained in absence of *Wp*-CFS.

To further assess the bacteriostatic behavior of the compound produced by *W. paramesenteroides* UFTM 2.6.1, time-kill experiments against *L. monocytogenes* strains were performed. At a concentration of 4x MIC, the time-kill curve differed significantly from the control group (absence of *Wp-*CFS) (*p* < 0.05): the bacterial cell population remained stable at approximately10^6^–10^7^ CFU/mL over the 72 h incubation period, with no evidence of bacterial regrowth (Figure 2). No differences were observed in time kill curves at 0.25x, 0.5x, and MIC value when compared to the control group (*p* > 0.05). These findings corroborated the MIC/MBC results, and support the conclusion that the compound produced by *W. paramesenteroides* UFTM 2.6.1 has bacteriostatic activity against *L. monocytogenes*.

### 3.2 Biofilm formation capacity by *Listeria monocytogenes* strains

**Figure 3:**
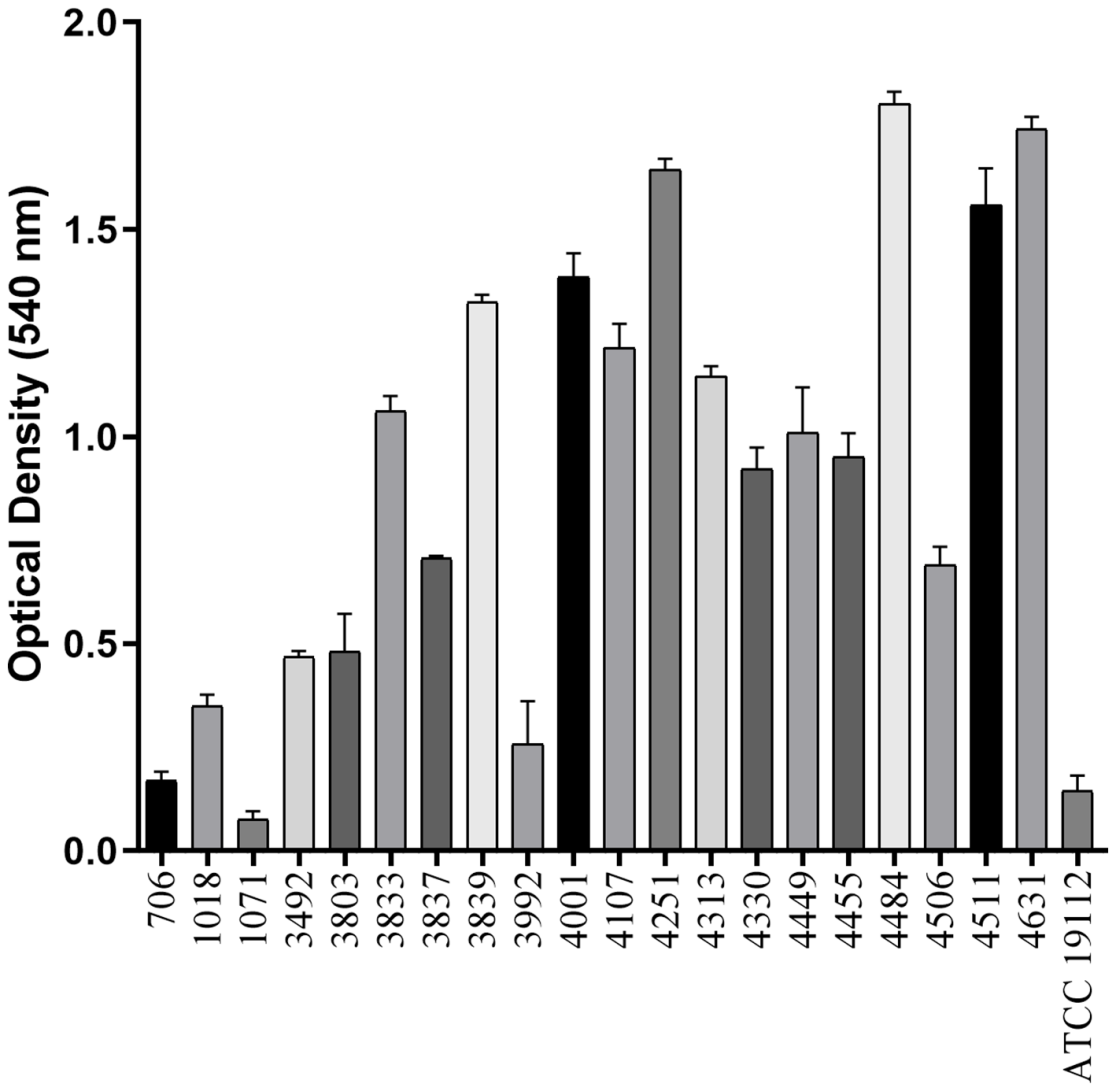
Total biofilm biomass of *Listeria monocytogenes* strains on polystyrene microplates. Bars represent the mean and standard deviations of three independent experiments.

Seventeen of the twenty-one strains of *L. monocytogenes* formed biofilm after incubation at 28°C for 72 h. The OD540 nm values obtained revealed the variability in biofilm biomass among the *L. monocytogenes* strains, with 14 strains (3833, 3837, 3839, 4001, 4107, 4251, 4313, 4330, 4449, 4484, 4511, 4455, 4506, and 4631) identified as strong biofilm formers, which were selected for further experiments (Figure 3).

### 3.3 *Weissella paramesenteroides*-cell-free supernatant reduces biofilm formation and motility of *Listeria monocytogenes* strains

**Figure 4:**
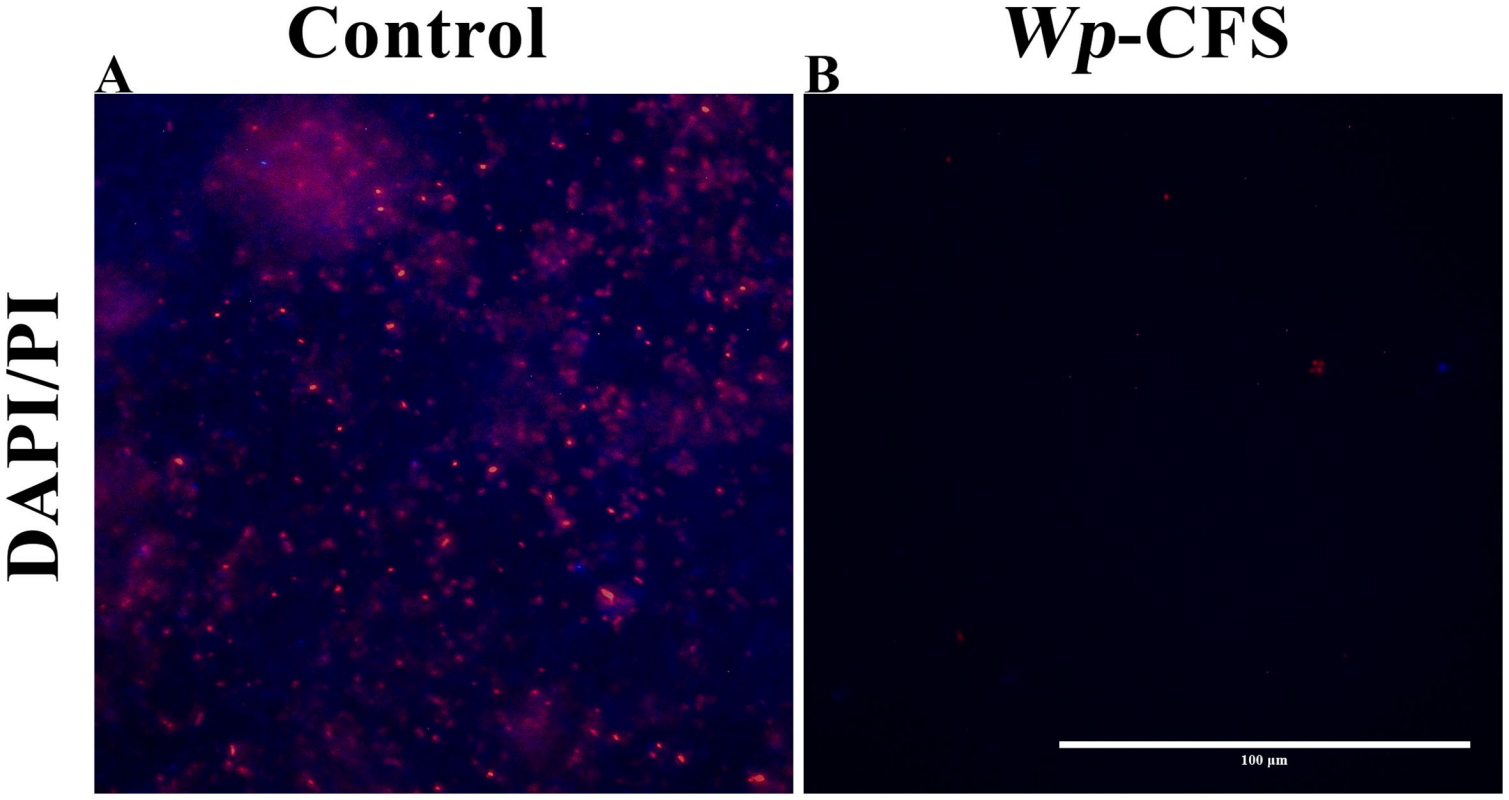
Fluorescence microscopy images of biofilm cells of *Listeria monocytogenes* strain 4455 on polystyrene microplates, either untreated (control; **A**) or exposed to *Weissella paramesenteroides*-cell-free supernatant (*Wp*-CFS; **B**). The biofilms were DAPI/PI stained (represented in blue and red on images, respectively). The images are representative of three biological replicates. The bar represents 100 μm.

A significant reduction in the development of biofilms by *L. monocytogenes* strains was observed upon the addition of *Wp*-CFS (Table 3; Figure 4). Concentrations equal to or above the MIC significantly reduced biofilm formation (up to 100% reduction for several bacterial strains) when compared to the control (non-treated biofilms). At 0.5x MIC (15.6 mg/mL), biofilm formation inhibition rates ranged from 59.8 to 89.5%. A more prominent effect of *Wp*-CFS was observed against strains 3833, 4330, 4455, and 4631, for which a reduction in biofilm formation was observed even at 0.125x MIC (3.9 mg/mL) (Table 3).

**Table 3:** Effect of *Weissella paramesenteroides*-cell-free supernatant (*Wp*-CFS; 0.125x to 4x MIC) in reducing biofilm formation by *Listeria monocytogenes in vitro* at 28°C for 72 h.

Strains	Biofilm formation reduction (%)					
	4x MIC	2x MIC	MIC	0.5x MIC	0.25x MIC	0.125x MIC
3833	100.0*	100.0*	92.4*	77.3*	66.0*	42.1*
3837	83.2*	81.3	78.7*	59.8*	31.8*	0.0
3839	100.0*	96.4*	90.3*	78.5*	36.5*	0.0
4001	100.0*	100.0*	88.6*	83.6*	41.9*	0.0
4107	100.0*	100.0*	83.9*	64.5*	8.9	8.8
4251	100.0*	99.2*	91.0*	89.2*	65.9*	27.6
4313	84.6*	82.5*	80.3*	65.8*	35.5*	0.0
4330	100.0*	96.9*	84.2*	89.5*	71.0*	51.2*
4449	100.0*	99.3*	76.7*	81.3*	64.6*	8.0
4484	99.9*	97.5*	92.5*	83.8*	55.0*	0.0
4455	100.0*	90.6*	83.1*	81.6*	70.1*	35.1*
4506	82.6*	82.1*	80.7*	61.3*	33.4*	0.0
4511	81.4*	92.2*	84.0*	83.8*	37.4*	0.0
4631	100.0*	100.0*	99.5*	76.9*	48.4*	43.5*

* indicate significant differences compared to the control group (biofilm formed in the absence of Wp-CFS), according to Dunnett’s test (*p* < 0.05).

In the presence of *Wp*-CFS at concentrations equal to or above the MIC value, a few remaining adhered *L. monocytogenes* cells were observed under fluorescence microscopy (Figure 4B), unlike the control, which presented a compact and mature biofilm (Figure 4A). This result confirms the anti-biofilm activity of *Wp*-CFS.

**Figure 5:**
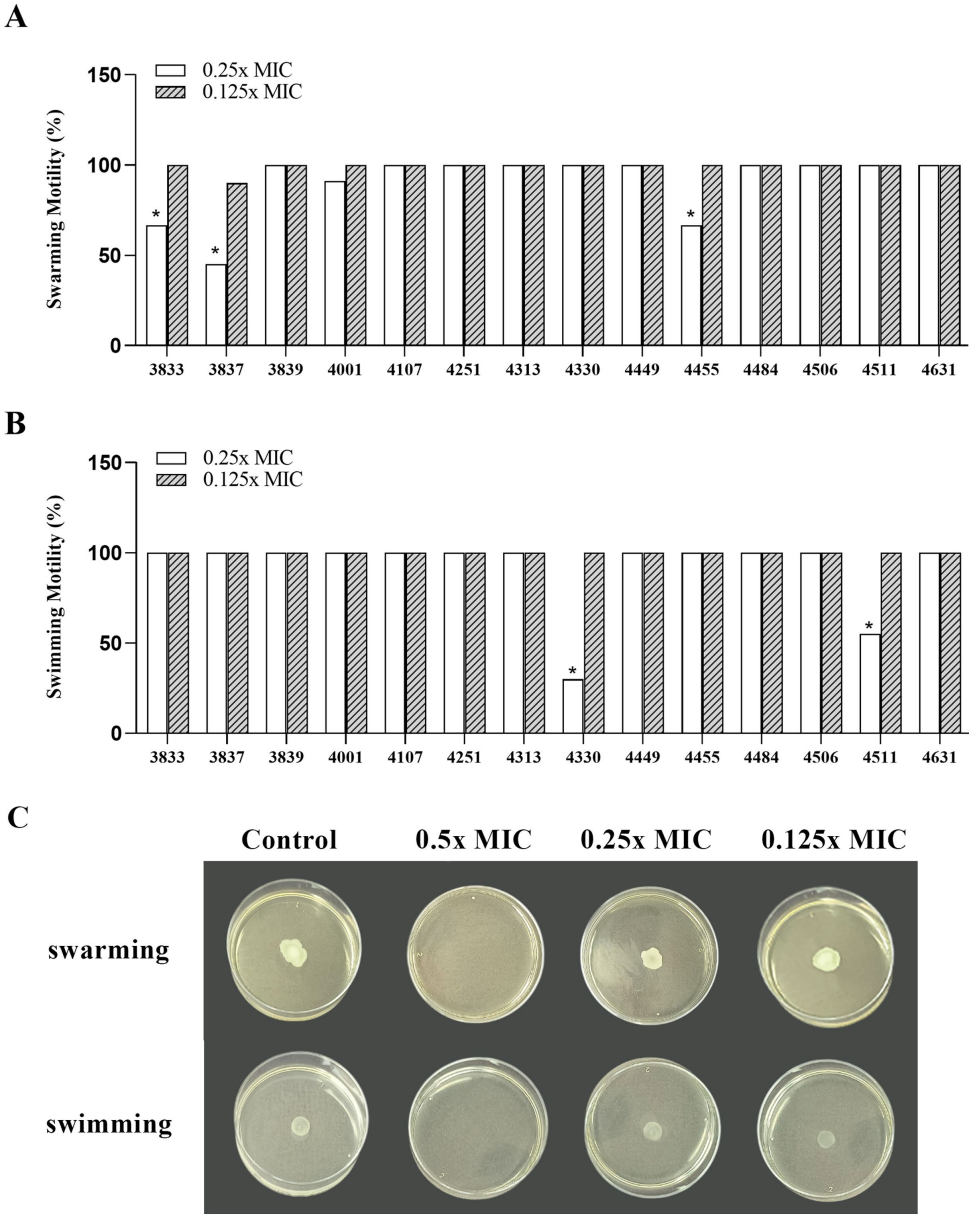
Efficacy of different concentrations of *Weissella paramesenteroides*-cell-free supernatant (*Wp*-CFS; 0.25x MIC and 0.125x MIC) in inhibiting motility ability of *Listeria monocytogenes*, at 28°C. **(A)** Swarming ability, **(B)** swimming ability, **(C)** representative images of the *Wp*-CFS activity on motility pattern of the *Listeria monocytogenes* strain 4455. Bars with an asterisk indicate significant differences compared to the control group (non-exposed to *Wp-*CFS), according to Dunnett’s test (*p* < 0.05).

Swimming motility refers to individual movement in liquid powered by rotating flagella, while swarming motility is a multicellular surface movement driven by rotating helical flagella and regulated by *quorum sensing*. In the presence of 0.25x MIC (7.8 mg/mL) of *Wp*-CFS, the swarming motility of *L. monocytogenes* strains 3833, 3837, and 4455 showed a significant reduction compared to non-exposed cells (33.3, 45.0, and 33.3%, respectively) (Figures 5A,C). Swimming motility of *L. monocytogenes* was not affected by the presence of 0.25x MIC of *Wp*-CFS, except for strains 4330 and 4511 (30.0 and 55.0%, respectively) (Figures 5B,C). At 0.5x MIC of *Wp*-CFS, no bacterial growth was observed, while lower concentrations (0.125x MIC) did not affect the motility patterns of the *L. monocytogenes* strains evaluated (Figure 5C).

### 3.4 *Weissella paramesenteroides*-cell-free supernatant modulates the expression of target genes in *Listeria monocytogenes* 4455

**Figure 6:**
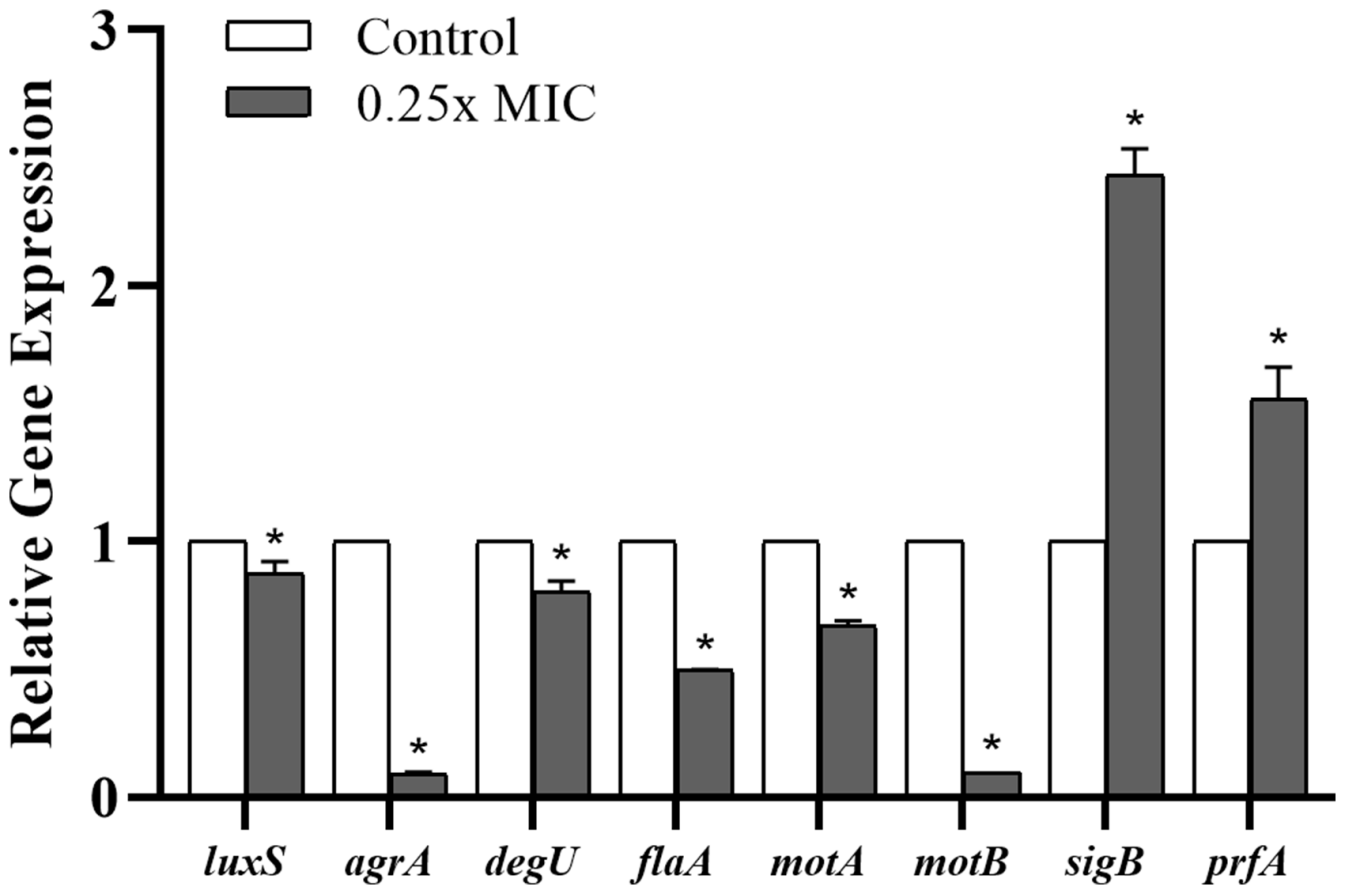
Relative gene expression of *Listeria monocytogenes* strain 4455 in response to exposure to 0.25x MIC of *Weissella paramesenteroides*-cell-free supernatant (*Wp*-CFS), at 28°C for 24 h. *Indicates statistical differences when compared to the control group (non-exposed to *Wp-*CFS) (*p* < 0.05). Mean values of three independent experiments are shown.

Considering the prominent effect of the *Wp*-CFS treatment on reducing biofilm formation and swarming motility of the *L. monocytogenes* strain 4455, this lineage was selected for transcriptional analysis of target genes related to QS, biofilm formation, and motility. Compared to untreated bacterial cells, the treatment with 0.25x MIC of *Wp*-CFS decreased the expression of the QS genes *agrA* and *luxS*. In addition, genes associated with biofilm formation, motility and flagella (*degU*, *flaA*, *motA*, and *motB*) were all significantly downregulated (*p* < 0.05) by *Wp*-CFS (Figure 6). On the other hand, the genes *sigB*, a global regulator of the stress response, and *prfA*, the main regulator of virulence factors in *L. monocytogenes* were upregulated (*p* < 0.05) in the presence of *Wp*-CFS (Figure 6).

## 4 Discussion

Bacteria of the genus *Weissella* are recognized to produce bioactive compounds with different mechanisms of action. Notable bioactive compounds produced by *Weissella* species include organic acids, hydrogen peroxide, diacetyl, bacteriocins, exopolysaccharides, antioxidants, vitamins, and short-chain fatty acids (Singh et al., 2024; Deatraksa et al., 2018; Yu et al., 2019; Kavitake et al., 2020; Månberger et al., 2020; Zhao et al., 2021; Fhoula et al., 2022; Hernández-Alcántara et al., 2022; Tuccillo et al., 2022; Lahmar et al., 2024). The diversity of these bioactive metabolites makes *Weissella* a promising candidate for applications in food preservation, the probiotic industry, and the development of new natural products with antimicrobial and therapeutic properties. Despite the well-recognized antimicrobial properties and beneficial effects in food fermentation, research into the specific quorum-quenching abilities of *Weissella* species is still underexplored. Most studies on this topic remain focused on other LAB genera, such as *Lactobacillus* and *Enterococcus*, which have been screened for the production of QQ molecules that interfere with QS systems of several pathogens (Chatterjee et al., 2017; Boopathi et al., 2017; Ham et al., 2018; Cui et al., 2020; Vadassery and Pillai, 2020; Qiao et al., 2021; Lv et al., 2021; Vasiee et al., 2022; Tomé et al., 2023; Shi et al., 2024).

Some studies have shown that *Lactobacillus* species produce molecules that reduce *L. monocytogenes* virulence, motility, biofilm formation, and/or can interfere with QS signaling (Moradi et al., 2019; Singh et al., 2020; Jara et al., 2020; Hossain et al., 2021; Kıran et al., 2021; Lee et al., 2021; Liu et al., 2022; Davares et al., 2022). In the present study, we demonstrate for the first time that *W. paramesenteroides* UFTM 2.6.1 may also produce QQ compounds, which have the potential to prevent biofilm formation, in addition to reducing motility and downregulating virulence gene expression in *L. monocytogenes*.

To investigate the effect of *Wp*-CFS on *L. monocytogenes*, 21 strains isolated from food and food processing sectors with four different serotypes were selected for this study. After confirming the antagonistic activity of *W. paramesenteroides* UFTM 2.6.1 on *L. monocytogenes* strains, both in solid and liquid media, and selection of the best biofilm-producing strains, we demonstrated the bacteriostatic effect of *Wp*-CFS through time-kill experiments. Those results substantiated the hypothesis that we were addressing a potential QQ compound produced by *W. paramesenteroides* UFTM 2.6.1, since the premise of a QQ molecule is to attenuate bacterial virulence without directly killing the target bacteria. It is important to highlight that QS assays should be performed in concentrations that do not interfere with bacterial growth, to avoid the interference of cell density differences in QS regulated phenotypes (Defoirdt et al., 2013; Santos et al., 2021).

The ability to form biofilm is an important feature associated with persistence and antimicrobial resistance among bacterial pathogens (Costerton et al., 1999; Donlan and Costerton, 2002; de la Fuente-Núñez et al., 2013a). *L. monocytogenes* biofilms can serve as a source of food contamination through the release of cells attached to their structure (dispersal phase), enabling the continuous contamination of food products in the production chain (Poimenidou et al., 2009; Miquel et al., 2016). Different compounds extracted from plants or microbial cultures have shown anti-biofilm effects against *L. monocytogenes* (Riedel et al., 2009; Zamani et al., 2017; Li et al., 2025; Rocha et al., 2019).

Even at lower concentrations, below the MIC value, *Wp*-CFS consistently reduced biofilm formation and motility, two well-known QS regulated phenotypes in *L. monocytogenes.* It should be noted that at concentrations higher than the MIC, the reduction in biofilm formation did not significantly differ from the reduction obtained at MIC. Moreover, an inhibition of *L. monocytogenes* growth was observed in motility assays when *Wp*-CFS was added at MIC value, which likely affected motility through a QS-independent mechanism.

According to the results obtained, it is likely that the anti-biofilm property of *Wp*-CFS could be due to reduced *L. monocytogenes* adhesion capacity in the early stages of biofilm development or preventing the switch to the biofilm phenotype. Our findings can be substantiated with a study conducted by Jamwal et al. (2019), which reported maximum biofilm inhibitory activity (>80%) of probiotic strains at initial stages of biofilm formation, whereas weaker activity was observed after prolonged incubation (48 h). Other studies (Gálvez et al., 2007; Winkelströter et al., 2014; Zhou et al., 2020; de la Fuente-Nunez et al., 2013b; Pimentel-Filho et al., 2014; Segev-Zarko et al., 2015; Ahn et al., 2018) reported that LAB supernatants or compounds produced by LAB, such as bacteriocins and exopolysaccharides, can interfere with the early events of biofilm formation by preventing bacterial cells adhesion to surfaces or to each other cells or by killing cells before they stably become part of the biofilm architecture.

To enlighten possible mechanisms underlying the anti-*Listeria* activity of *WP-*CFS, we verified the gene expression of several target genes in *L. monocytogenes.* Our results showed that the exposure to a sub-inhibitory concentration of *Wp*-CFS (0.25x MIC) reduced the expression of both *luxS* and *agrA*, which, consequently, could impair QS communication in *L. monocytogenes*. Disruption of a single component within the QS pathway frequently results in the downregulation of QS related genes and subsequent inactivation of the QS mechanism (Galié et al., 2018).

*LuxS* is the enzyme responsible for the biosynthesis of the signal molecule AI-2. LuxS/AI-2 is an important QS system, present in both Gram-negative and Gram-positive bacteria, and has therefore been proposed as a universal signal enabling interspecies cell–cell communication. In *L. monocytogenes*, intraspecies communication (Skandamis and Nychas, 2012; Kocot and Olszewska, 2017) occurs via the Agr system, which consists of the four-gene operon *agrBDCA*. AgrD, the precursor peptide, is processed into an active signaling molecule called autoinducing peptide (AIP) by AgrB, and the AIP is released outside the cells; once the AIP reaches critical concentration, it activates the two-component system AgrC/AgrA (receptor-histidine kinase/response regulator) (Lina et al., 1998).

In some pathogenic bacteria, LuxS was found to be involved in biofilm formation (Sela et al., 2006). However, the contribution of the *luxS* gene to biofilm formation in *L. monocytogenes* remains controversial. Belval et al. (2006) found that the *luxS* mutant gene in *L. monocytogenes* resulted in an increase in biofilm formation at 25°C. On the other hand, Bonsaglia et al. (2014) noted that *L. monocytogenes* strains with the *luxS* gene did not consistently produce biofilms. Zhang et al. (2022) found that the amount of biofilm formation was reduced in the *luxS* gene deletion strain of *L. monocytogenes*. In a study conducted by Gao et al. (2024), higher mRNA levels of *agrA*, *agrB*, *agrC*, and *luxS* in *L. monocytogenes* strains were not directly correlated with high biofilm capacity *in vitro.* Those conflicting findings regarding the role of *luxS* in biofilm formation by *L. monocytogenes* can be explained, at least in part, by strain-specific responses or experimental differences.

According to Yang et al. (2024), the expression level of the *agrA* gene in *L. monocytogenes* strains with strong biofilm-forming capacity is up-regulated after biofilm formation, unlike poor biofilm-forming strains, in which no differential expression is generally observed. This finding demonstrated that *agrA* gene plays a positive regulatory role in the biofilm formation process. In this sense, the down-regulation of *agrA* may reduce the development of *L. monocytogenes* biofilms (Rieu et al., 2007; Riedel et al., 2009), which was clearly demonstrated in the presence of *Wp*-CFS.

Flagella are recognized as key factors in facilitating the initial contact of the bacterial cell with surfaces in the early stages of biofilm formation (Haiko and Westerlund-Wikström, 2013; Fan et al., 2020; Benyoussef et al., 2022; Coloma-Rivero et al., 2022; Vilas Boas et al., 2024). It has been suggested that flagellum-mediated motility may assist in overcoming repulsive forces at the surface, thereby enabling initial attachment (Benyoussef et al., 2022). Swarming motility is a specialized form of motility on solid surfaces, dependent on extensive flagellation, cell–cell contact, and driven by QS, while swimming motility refers to the movement of individual bacteria, which involves the rotation of flagella to propel the cell through liquid environments.

The presence of sub-inhibitory concentrations of *Wp*-CFS reduced not only swarming motility but also the expression levels of the motility-associated genes *degU, flaA*, *motA* and *motB*, which additionally provides insights into the mechanism of action of *Wp*-CFS in disrupting QS-system and reducing *in vitro* biofilm formation by *L. monocytogenes*. The *degU* gene encodes a response regulator in *L. monocytogenes* that regulates the expression of flagellin and motility genes (Williams et al., 2005; Zhu et al., 2023). The *flaA* gene encodes flagellin A (FlaA), the structural subunit of the flagellum, while the *motA* and *motB* genes encode the flagellar motor proteins MotA and MotB, respectively, which control flagellar movement (Gründling et al., 2004; Casey et al., 2014).

The transcriptional activator PrfA (positive regulatory factor A) has been identified in *L. monocytogenes*, and it plays a crucial role in the regulation of most genes associated with *L. monocytogenes* pathogenesis, including the virulence island 1 (LIPI-1) genes (Gaballa et al., 2019; de las Heras et al., 2011). Sigma factor B (*sigB*) is the global regulator of the stress response, and it has been reported to be involved in biofilm formation and in the adaptation process of *L. monocytogenes* strains to low-temperature environments (Liu et al., 2021; Hu et al., 2007; Zhou et al., 2020; Vázquez- Armenta et al., 2020). The transcription of both the *prfA* and *sigB* genes was upregulated in *L. monocytogenes* in the presence of 0.25x MIC of *Wp*-CFS. This result may indicate an attempt by the bacterial cell to activate pathogenesis- and stress-related genes in order to resist and survive the external stimulus caused by the presence of *Wp*-CFS.

A similar result was observed in a study conducted by Huang et al. (2020), in which the photodynamic inactivation treatment upregulated the expression of the *prfA* while significantly reducing the adhesion ability of *L. monocytogenes* biofilms. In contrast, Masebe and Thantsha (2022) found that the expression of the *prfA* gene was significantly reduced in the presence of CFS of LAB strains, with a consequent inhibition and/or dispersion of *L. monocytogenes* biofilms. In a study conducted by Liu et al. (2021), the natural compounds cinnamaldehyde and eugenol down-regulated the transcription of the *prfA* and *sigB*, a result associated with the inhibition of *L. monocytogenes* biofilm formation.

The downregulation of key regulatory (*luxS*, *agrA*, *degU*) and motility-associated genes (*flaA*, *motA*, *motB*) in the presence of *Wp-CFS*, alongside its ability to significantly reduce *Listeria monocytogenes* biofilm formation in vitro, suggests that *Wp-CFS* interferes with upstream regulatory systems involved in QS and environmental signal processing. A plausible hypothesis for quorum-quenching activity of *Wp-CFS* is that it disrupts QS-mediated regulatory cascades either by reducing QS signal molecules (e.g., AI-2) or by antagonizing signal perception. The repression of *luxS*, a gene central to AI-2 biosynthesis, and *agrA*, a critical component of the Agr QS system, implies interference at both signal production and response levels. This disruption likely affects intermediate regulators such as *degU*, which ultimately downregulating downstream targets involved in motility and biofilm formation. Since motility is often a prerequisite for initial surface colonization and biofilm development, the coordinated repression of motility and QS genes by *Wp-CFS* provides a plausible mechanistic explanation for its antibiofilm activity. Together, these results support a model in which the QQ activity of *Wp-CFS* impairs QS-regulated phenotypes in *L. monocytogenes*, offering a promising strategy for attenuating virulence and persistence of this pathogen.

## 5 Conclusion

This study presents a novel contribution to the field by demonstrating the previously unreported potential of *Weissella paramesenteroides* in disrupting the QS mechanism of the foodborne pathogen *Listeria monocytogenes*. This discovery broadens the functional scope of *W. paramesenteroides* UFTM 2.6.1 as a beneficial microorganism, highlighting its promise as a biocontrol agent in food-related and biomedical sector. The identification of compounds with anti-biofilm and anti-virulence properties supports its potential application.

Further investigations are underway to identify the active constituents of *Wp-CFS*, to characterize the molecular mechanisms underlying the QQ activity. Additionally, future studies should focus on validating the effects of *Wp-CFS* in complex environments and in animal models. These insights will be pivotal for advancing its practical application in food bioprotection, to ensure food safety.

## Data Availability

The original contributions presented in the study are publicly available. This data can be found at: https://www.ebi.ac.uk/ena/browser/view/SAMEA115664826.
